# Editors’ Preamble to *The Journal of Cardiovascular Aging*

**DOI:** 10.20517/jca.2021.01

**Published:** 2021-04-27

**Authors:** Ali J. Marian, Babken Asatryan, Roberto Bolli, Sirisha M. Cheedipudi, Naranjan S. Dhalla, Toren Finkel, Nikolaos G. Frangogiannis, Priyatansh Gurha, Juan Carlos Izpisua Belmonte, Joshua M. Hare, Kui Hong, Lorrie A. Kirshenbaum, Richard T. Lee, Massoud A. Leesar, Peter Libby, Rosalinda Madonna, Sherif F. Nagueh, Robert Roberts, Anthony Rosenzweig, Leila Rouhi, Junichi Sadoshima, Mark Alan Sussman, George E. Taffet, Hirofumi Tanaka, Daniele Torella, Yibin Wang, Dao Wen Wang

**Affiliations:** 1 Center for Cardiovascular Genetics, Institute of Molecular Medicine and Department of Medicine, University of Texas Health Sciences Center at Houston, Houston, TX 77030, USA; 2 Department of Cardiology, Inselspita, Bern University Hospital, University of Bern, Bern 3010, Switzerland; 3 Department of Medicine, Division of Cardiovascular Sciences, University of Louisville, Louisville, KY 40292, USA; 4 Department of Physiology and Pathophysiology, Max Rady College of Medicine, University of Manitoba, Winnipeg MB R2H 2A6, Canada; 5 Aging Institute, University of Pittsburgh and University of Pittsburgh Medical Center, Pittsburgh, PA 15219, USA; 6 Department of Medicine (Cardiology), Albert Einstein College of Medicine, Bronx, NY 10461, USA; 7 Gene Expression Laboratory, Salk Institute for Biological Studies, La Jolla, CA 92037, USA; 8 Interdisciplinary Stem Cell Institute and Department of Medicine, University of Miami Miller School of Medicine, Miami, FL 33136, USA; 9 Department of Cardiovascular Medicine and Jiangxi Key Laboratory of Molecular Medicine, The Second Affiliated Hospital of Nanchang University, Nanchang 330006, Jiangxi, China; 10 Department of Stem Cell and Regenerative Biology and the Harvard Stem Cell Institute, Harvard University, Cambridge, MA 02138, USA; 11 Department of Medicine, Brigham and Women’s Hospital and Harvard Medical School, Boston, MA 02115, USA; 12 Division of Cardiology, University of Alabama-Birmingham, Birmingham, AL 35233, USA; 13 Department of Surgical, Medical, Molecular and Critical Area Pathology, Institute of Cardiology, University of Pisa, Pisa 56124, Italy; 14 Department of Cardiology, Methodist DeBakey Heart and Vascular Center, Houston, TX 77030, USA; 15 Department of Medicine, Dignity Health at St. Joseph’s Hospital and Medical Center, Phoenix, AZ 85013, USA; 16 Department of Medicine, Massachusetts General Hospital, Harvard Medical School, Boston, MA 02115, USA; 17 Department of Cell Biology and Molecular Medicine, Rutgers New Jersey Medical School, Newark, NJ 07103, USA; 18 Department of Biology, San Diego State University, San Diego, CA 92182, USA; 19 Department of Medicine, Baylor College of Medicine, Houston, TX 77030, USA; 20 Department of Kinesiology and Health Education, The University of Texas at Austin, Austin, TX 78712, USA; 21 Department of Experimental and Clinical Medicine, Magna Graecia University, Catanzaro 88100, Italy; 22 Departments of Anesthesiology, Physiology, and Medicine, Cardiovascular Research Laboratories, David Geffen School of Medicine, University of California, Los Angeles, CA 90095, USA; 23 Department of Internal Medicine, Tongji Hospital, Tongji Medical College, Huazhong University of Science and Technology, Wuhan 430030, Hubei, China

Biological aging, defined as a gradual decline of the biological/physiological processes that sustain life, is a constitutive element of living and hence, is inescapable. Not only is aging unavoidable but so is its obligatory outcome of death. Naturally, the humans have fantasized about means to avoid aging and maintaining youth since the birth of civilization. The desire to stay young has been universal amongst cultures and epochs, as depicted in the ancient mythology, arts, and literatures. Hebe, the cup-bearer to the Gods and the daughter of Zeus and Hera, was the Goddess of Eternal Youth who had the power to restore eternal youth to aging Gods. In Charles Gounod’s opera Faust, the aged philosopher Dr. Faust morphed into a handsome young man by drinking the elixir of youth offered by Mephistopheles in exchange for his place in hell. In Oscar Wilde’s “The Picture of Dorian Gray”, Dorian sought eternal youth by selling his soul in pursuit of hedonistic life. Despite such a strong yearning to prevent aging, the progress has been slow to come. In “The End of Science”, Horgan^[1]^, an eminent science writer, argues that aging is inextricably integrated into the biology of all organisms and therefore, is unconquerable. Not dissuaded by Horganism, Sir Maddox^[2]^, the legendary editor of *Nature*, in “What Remains to Be Discovered” argues that the flow of fundamental discoveries is ceaseless. Consequently, scientific advances will ultimately enable extension of the healthspan and slowing the aging process, if not halting it. The remarkable progresses in molecular genetics and biology have just begun to impact the practice of medicine, as best illustrated by COVID-19. It is quite remarkable that within a year or so after the first case of COVID-19 was reported, several highly effective vaccines were developed and became available for mass vaccination. Thus, the task of scientists is to discover and never claim to have the final say in any scientific matter. The mission of scientific journals is to disseminate such discoveries.

Biological aging is defined very broadly as a series of processes that start from the very beginning of the life of a cell or an organism. While the observational findings might be model specific or subject to the experimental conditions, fundamental discoveries would be expected to have universal implications. Consequently, a discovery that elucidates the fundamental basis of aging or senescence, whether observed in the yeast or in a mammalian model organism, will be pertinent to cardiovascular system as well. The cardiovascular system is a prime target of biological aging, and its involvement is a major determinant of the inevitable outcome of aging. Aging burdens the coronary arteries with atherosclerotic plaques and calcifications, degenerates the valves rendering them malfunctional, thins the contractile units of the myocytes impairing their relaxation and ability to utilize mitochondrial energy, expands and thickens the myocardial collagenous network reducing ventricular compliance and disrupting impulse conduction, distorts the aorta and blood vessels making them non-complaint, and makes cardiovascular system less resilient to stress and injury with diminishing capacity of repair and regeneration. To counter the process of cardiovascular aging, it is essential to discover the fundamental mechanisms that govern aging, whether at the cellular or organismal level, make astute clinical observations, and intervene to prevent, attenuate, and reverse the processes. It is also necessary and timely to establish a desirable platform that enables the clinicians and scientists to disseminate their seminal discoveries and observations pertaining to cardiovascular aging. *The Journal of Cardiovascular Aging (JCA)* provides such a dedicated platform for rapid dissemination of clinical, translational, and basic scientific discoveries related to cardiovascular aging. The *JCA* is the first journal specifically dedicated to cardiovascular aging. It fills the niche in this field of increasing importance. The journal is a member of a member of Committee on Publication Ethics (COPE) and supports Gold Open Access, the latter provides access to full content of the articles free of charge.

The editors are committed to developing the *JCA* as the premier, most authoritative, and entirely transparent international platform for dissemination of state-of-the-art basic, translational, and clinical studies in aging and cardiovascular disease. The *JCA* aims to publish discoveries that pertain to all aspects of aging and cardiovascular disease. Aging is considered in a broader view and considered a process that begins with the birth of a cell and continues till the death of the organism. Therefore, all aspects of basic and clinical sciences related to aging within the context of cardiovascular disease are considered to be within the scope of the journal.

The categories of articles that will be considered for publication in the *JCA* are listed in the instructions to the authors and the editorial policy is published at the journal website (https://cardiovascularaging.com/). Briefly, the primary focus of the journal is to publish original research articles, whether in full-length article or brief report format, that provide novel mechanistic insights into cardiovascular aging. Studies confirming, validating, or challenging existing dogmata, whenever providing unequivocal findings, are considered to be within the scope of the journal. Typically, original research articles will be accompanied by editorials to highlight strengths and weaknesses of the articles and surmise the significance of the findings. While the focus is on publication of original scientific discoveries, the journal will also publish state-of-the-art review manuscripts on topics of broad interest to the readership of the journal. Furthermore, the journal will invite the leading authorities to write commentaries on impactful discoveries published elsewhere, as well as perspectives. Case reports and case series, as well as unique medical images will be considered. The editors are committed to enhancing the career development of early-stage investigators through various platforms, including profiling the young investigators in the journal. Finally, young investigators have been recruited to function as academic assistant editors in scanning the scientific journals and summarizing new and significant findings related to aging in the category of News.

Dissemination of scientific discoveries is essential for advancing the biomedical sciences. The four key elements of the biomedical publishing are the authors, the referees (reviewers), the editors, and the publisher.

## The authors:

The authors, fully aware of the dictum “publish or perish”, which is ingrained in the academic culture, aim to publish their findings in the most prestigious journals in order to get recognition within and outside of their organization. The pressure must not obscure the main purpose of scientific publications, which is to disseminate discoveries with the ultimate goal of favorably impacting the lives of patients and preventing illnesses. The primary responsibility of the authors is to present their observations in the most lucid and clear fashion, allowing the data to speak for themselves, without embellishment or excessive emphasis. The success of a manuscript is partly based upon the ability of the authors to present their findings in the most succinct and lucid manner. It is essential to minimize, and if possible, to eliminate all known biases when setting up the experiments to test the hypothesis. It is easy to ignore those ugly data that slay a beautiful hypothesis. This temptation must be avoided. Likewise, it is crucial that the authors do not twist the data to fit into their hypothesis, instead of fitting the hypothesis into the data, as the latter was Sherlock Holmes’ mantra in solving the puzzles.

## The reviewers:

The rapid pace of advances in biomedical sciences and the increased number of the scientific papers has necessitated prioritization of manuscripts for publication, based on their scientific contents. The expanded depth and breadth of biomedical research have resulted in an increased reliance on evaluations of manuscripts by the external and typically anonymous referees. Peer-review approach, despite its limitations, has emerged as the key component of judging the merit of the scientific manuscripts. The reviewers are usually impartial judges who render their evaluations pro bono and do not benefit from much recognition for their hard work. They are exceedingly generous with their time and in offering scientific guidance to the authors.

Reviewers are committed to excellence in scientific publications and are seldom biased. Such departures from normality are easily recognized by the editors and properly handled. On occasion, the reviewers place an excessive burden on the authors to conduct experiments that are often tangential to the original discoveries and provide only incremental data. The point was elegantly expressed by Ploegh^[3]^ in a commentary in *Nature*, entitled “End the wasteful tyranny of reviewer experiments”. The editors are actively engaged in assessing the scientific content of the manuscripts as well as the reviewers’ comments with the objective of increasing the scientific quality of the manuscript, the robustness of the data, and the rigor of the findings, while eliminating unnecessary experiments.

## The editors:

The primary objective of the editors is to disseminate advances in cardiovascular sciences by publishing the best and most robust research related to aging. The editors have the ultimate responsibility in making the final disposition of the manuscripts, while functioning as active referees who carefully analyze not only the comments of the external reviewers but also the scientific content of the manuscript. The editors have the responsibility of making decisions about accepting or rejecting a manuscript based on the overall standing of the paper as compared to others being processed for publication in the journal.

Accessibility of the editors and a close rapport between the editors and the authors are the crux of enticing submission of top scientific discoveries to the journal. The editors will encourage direct pre-submission inquiries and provide a fair, efficient, and transparent direction to the authors. The editors are committed to providing timely review, processing of the manuscripts, and their publications.

## The publisher:

The publisher provides the platform for the journal to disseminate the scientific discoveries and assists the editors in managing all aspects of the scientific publishing, ranging from submission of articles to their publications. The publisher honors the total independence of the editors and does not interfere with the scientific content of the journal. Of course, the main objective of most, if not all, publishers is financial gain. The sage publisher realizes that the best way to achieve this objective is to provide a robust platform for dissemination of the most authentic scientific information to the benefit of the society and consequently, to the benefit of the publisher. Accordingly, the publication fee for the articles published in the *JCA* will be waived until the journal is indexed in Web of Science. The publisher also provides manuscript editing services for free. Finally, the publisher along with the editors will publicize the journal and its content in various social media and the scientific meetings.

In conclusion, the primary responsibility of authors, referees, editors, and the publisher is to disseminate the most robust data in cardiovascular sciences with the ultimate goal of improving the lives of our aging population and preventing diseases. Despite the best efforts by all involved, the process of publishing scientific papers is not without its shortcomings. The editors will strive to improve the process to the complete satisfaction of all parties involved, a task that requires tremendous efforts by all major constituents; including the authors, referees, editors, and the publisher. The editors of this journal have strong track records of accomplishments not only in their own scientific research fields but also with respect to their scientific integrity and dedication to furthering scientific advances.
